# Sources of individual variability: miRNAs that predispose to neuropathic pain identified using genome-wide sequencing

**DOI:** 10.1186/1744-8069-10-22

**Published:** 2014-03-19

**Authors:** Kiran Kumar Bali, Michael Hackenberg, Avigail Lubin, Rohini Kuner, Marshall Devor

**Affiliations:** 1 Pharmacology Institute, Medical Faculty Heidelberg, Heidelberg University, Im Neuenheimer Feld 366, Heidelberg 69120, Germany; 2 Department of Genetics, Faculty of Sciences, University of Granada, Granada 18071, Spain; 3 Department of Cell & Developmental Biology, Institute of Life Sciences and Center for Research on Pain, The Hebrew University of Jerusalem, Jerusalem 91904, Israel

**Keywords:** Dorsal root ganglion, miRNA-seq, Neuropathic pain, Rat, Regulation, Strategy

## Abstract

**Background:**

We carried out a genome-wide study, using microRNA sequencing (miRNA-seq), aimed at identifying miRNAs in primary sensory neurons that are associated with neuropathic pain. Such scans usually yield long lists of transcripts regulated by nerve injury, but not necessarily related to pain. To overcome this we tried a novel search strategy: identification of transcripts regulated differentially by nerve injury in rat lines very similar except for a contrasting pain phenotype. Dorsal root ganglia (DRGs) L4 and 5 in the two lines were excised 3 days after spinal nerve ligation surgery (SNL) and small RNAs were extracted and sequenced.

**Results:**

We identified 284 mature miRNA species expressed in rat DRGs, including several not previously reported, and 3340 unique small RNA sequences. Baseline expression of miRNA was nearly identical in the two rat lines, consistent with their shared genetic background. In both lines many miRNAs were nominally up- or down-regulated following SNL, but the change was similar across lines. Only 3 miRNAs that were expressed abundantly (rno-miR-30d-5p, rno-miR-125b-5p) or at moderate levels (rno-miR-379-5p) were *differentially* regulated. This makes them prime candidates as novel PNS determinants of neuropathic pain. The first two are known miRNA regulators of the expression of Tnf, Bdnf and Stat3, gene products intimately associated with neuropathic pain phenotype. A few non-miRNA, small noncoding RNAs (sncRNAs) were also differentially regulated.

**Conclusions:**

Despite its genome-wide coverage, our search strategy yielded a remarkably short list of neuropathic pain-related miRNAs. As 2 of the 3 are validated regulators of important pro-nociceptive compounds, it is likely that they contribute to the orchestration of gene expression changes that determine individual variability in pain phenotype. Further research is required to determine whether some of the other known or predicted gene targets of these miRNAs, or of the differentially regulated non-miRNA sncRNAs, also contribute.

## Background

Neuropathic pain phenotype varies greatly among individuals even when the underlying pathology is identical. An explanation of this variability could advance our understanding of chronic pain and perhaps lead to better therapeutic options. A key link between nerve pathology and pain is the response of primary afferent neurons to axotomy. Nerve trauma induces ongoing pain and hypersensibility by virtue of massive changes in gene expression that it triggers. Prominent among the resulting functional outcomes are the emergence of electrical hyperexcitability and phenotypic switching of neurotransmitter content [1]. The extent of these changes varies from individual to individual. We have tested the hypothesis that individual differences in neuropathic pain response are associated with the pattern of up- and down-regulation of gene expression. The experimental platform used was a pair of rat lines, HA and LA, that have similar genetic background and pain response at baseline, but radically different pain behavior following peripheral nerve injury [2,3]. Within each line individuals are inbred and hence genetically identical. But across lines a program of genetic selection resulted in a marked heritable difference in predisposition to pain. Thus, the lines are experimental surrogates for two individuals with contrasting pain response.

The inter-line contrast in neuropathic pain behavior in HA and LA rats is accompanied by appropriately contrasting changes in excitability and neurotransmitter profile [4,5]. Genetic analysis has attributed these differences largely to polymorphisms at a single quantitative trait locus (QTL; [6-8]. It is likely, therefore, that the line-specific pain phenotypes are due to differential regulation of gene expression following axotomy. However, given the complexity of pain behavior, it seems doubtful that regulation of a single gene product is to blame. More likely is a heritable element capable of controlling the expression of an entire constellation of functionally-related genes. This capability is inherent to microRNAs (miRNAs), small non-protein-coding RNAs known to play an important role in development and disease by post-transcriptional down-regulation of entire sets of target mRNAs [9-11]. Evidence supporting this hypothesis includes the facts that miRNAs are up- and down-regulated in sensory neurons in animal models of neuropathy, that certain salient miRNAs are prominently expressed in nociceptors and that pain is suppressed in transgenic mice in which global miRNA expression in nociceptors was selectively blocked [12-20]. This evidence, however, is not specific to pain. For example, the types of nerve trauma used to induce chronic pain also trigger changes in cell metabolism and transport, nerve regeneration and apoptosis.

Our rat lines provide a strategy for spotlighting those changes that are particular to pain. Specifically, we carried out a genome-wide search for nerve injury-induced changes in miRNA expression using both the pain-prone (HA) and the pain-resistant (LA) line. Since the lines were established by selection for neuropathic pain phenotype, unrelated processes such as regeneration are not expected to be line-specific. Following this strategy we asked whether there are any miRNAs, or other potentially regulatory small non-coding RNAs (sncRNAs), whose expression is *differentially* regulated in association with pain phenotype. Such genetic elements are likely to play a direct role in neuropathic pain physiology.

## Results

### Unique sequences and miRNAs in the rat DRG

**Figure 1:**
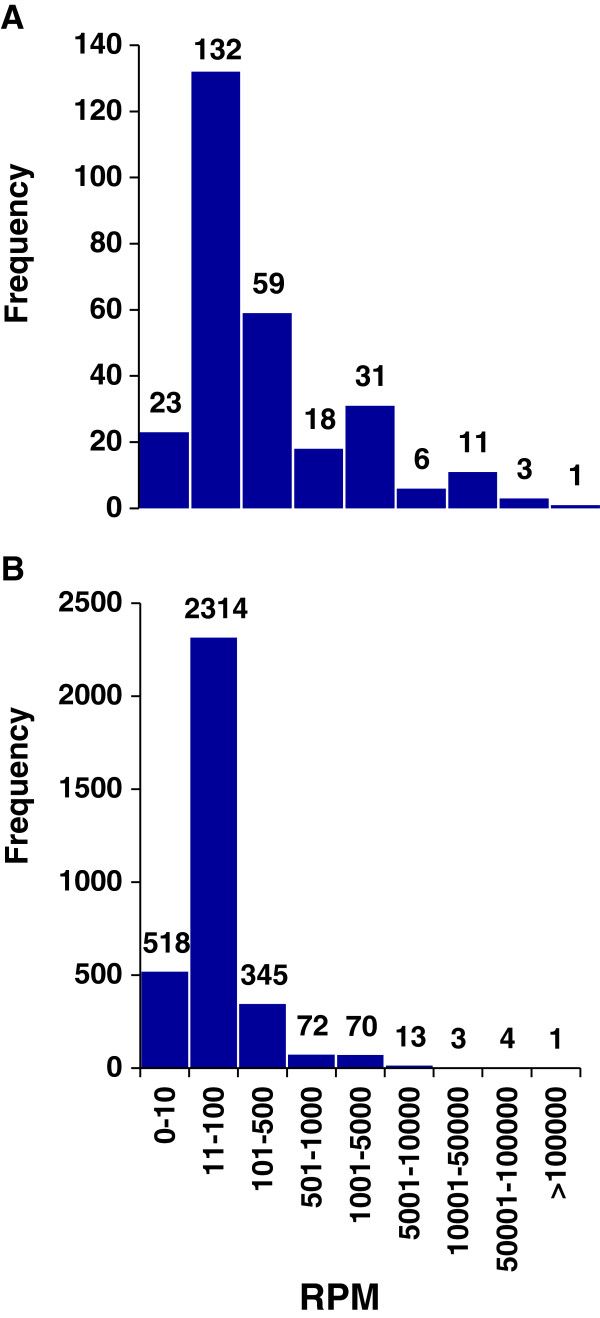
**Read counts of small non-coding RNAs in the rat DRG are skewed to smaller values. (A)** Mature miRNAs (n = 284). **(B)** Unique sequences of miRNAs and other sncRNAs. RPM values are based on the three replicate pools of the LA_sham experimental group (total n ~35 × 10^6^ reads). Distributions for the other three experimental groups were very similar.

After editing, mapping and excluding rare sequences, 3340 unique “reads level” single-stranded RNA sequences 16–41 nt in length were found that were expressed at RPM ≥10 (Reads Per Million) in at least 3 of the 12 biological pools (3 pools each for HA_sham, HA_SNL, LA_sham, LA_SNL). These included both miRNA isoforms and other sncRNAs. The overall number of such reads per pool was ~12.3 × 10^6^ with a remarkably uniform across-pools average (RPM = 271 ± 4). Within pools, however, there was a broad scatter of abundances with RPMs highly skewed towards the low end of the range (Figure 1A). The most abundant unique sequence had an RPM = 139,299 ± 43,970 across pools; for the least abundant, RPM = 6 ± 6.

MiRBase-v19 recognizes a total of 723 mature miRNA in the rat and many more in other species. Of these annotated miRNAs, 273 (38%) were detected at criterion abundance in our DRGs. In addition, 11 novel miRNAs, not previously reported in the rat (see below), passed threshold yielding a total of 284 mature miRNAs in the analysis. The overall abundance of mature miRNA reads in each pool was ~3.6 × 10^6^, about 30% of all reads. Total reads per miRNA identifier name averaged 12,388 ± 2,337 across pools. As for unique sequence reads, RPM values of the individual miRNAs filled a broad range across pools (1 to 523,440), again highly skewed towards lower values (Figure 1B). Additional file 1: Table S1 lists the 50 most abundantly expressed miRNA species in the rat DRG. No miRNAs were found to be expressed in one of the two rat lines but not the other, and none appeared *de novo*, or disappeared completely, after nerve injury. However, relative abundances differed somewhat between HA and LA rats at baseline, and abundances of some were significantly altered by SNL nerve injury.

### Line differences in basal expression of short regulatory RNAs

**Figure 2:**
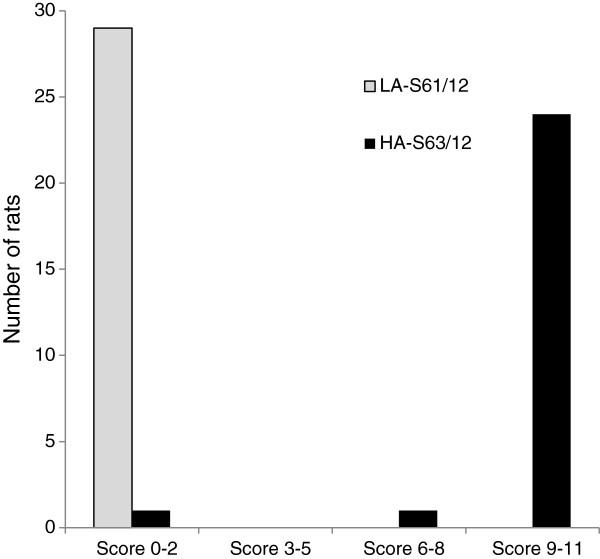
**Distribution of autotomy scores in HA and LA rats from the same generations as the rats used for miRNA sequencing.** The scoring protocol is detailed in [2].

Pain behavior was very different in HA and LA rats despite their similar genetic background [3], with HA animals achieving much higher autotomy scores than LA (Figure 2). Consistent with their common ancestry we identified only two miRNAs that were expressed at significantly different baseline levels in HA_sham and LA_sham pools. These were rno-miR-184 and rno-miR-6325, expressed 9.5-fold and 2.8-fold higher in HA than LA rats, respectively. Both occurred at low abundance (RPM = 22.7 and 16.1, respectively in HA). For the remaining 282 miRNAs (99.3%) FDR corrected p-value was >0.9. None of the 3340 reads-level unique sequences emerged as being differentially expressed at baseline after FDR correction.

### Effect of SNL nerve injury (*SNL-reg*)

Overall, about half of the 284 mature miRNAs in both HA and LA rats were nominally up-regulated following nerve injury (53.9% HA, 63.4% LA by subtraction method; 50.3% HA, 63.0% LA by log2 ratio). p-Values were significant (p < 0.05) before correction for multiple testing in n = 23 (HA) and n = 35 (LA). These are listed in Additional file 1: Table S2. However, only one remained significant after FDR correction for multiple testing (rno-miR-449c-5p). This result differs markedly from full-length mRNAs in which >10% of all expressed genes, >2,000, are significantly up- or down-regulated by 3 days after nerve injury [21-23]. The one exceptional miRNA was rare in both lines (mean RPM = 12.2 (HA) and 11.4 (LA), sham pools) and the degree of regulation did not differ between the lines (fold-change = 3.85 (HA) and 3.19 (LA); p = 0.85).

### Differential regulation of miRNAs following nerve injury (*diff-reg*)

**Figure 3:**
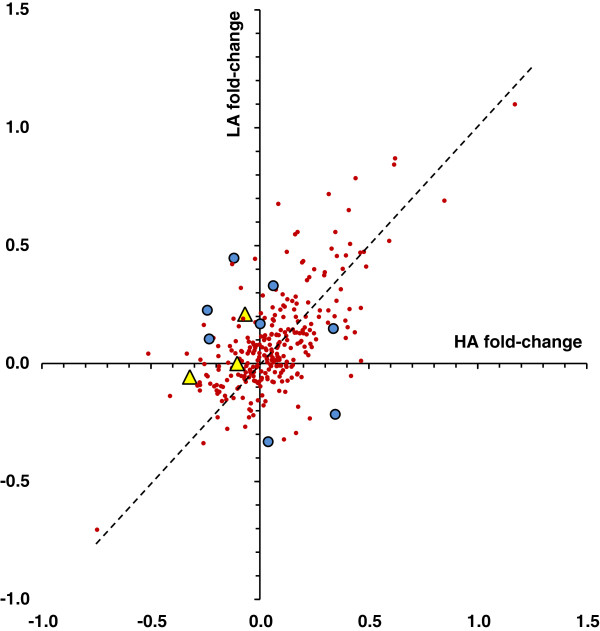
**SNL nerve injury induced roughly equal up- and down-regulation (*SNL-reg *) of miRNAs in DRGs of HA and LA rats (small red dots).** Symbols to the right of, and above, the axes indicate nominal upregulation. The few miRNAs that were differentially regulated (*diff-reg*) are indicated by yellow triangles (rno-miR-30d-5p, rno-miR-125b-5p and rno-miR-379-5p) or large blue dots. Regulation was calculated using the subtraction method (SNL-sham/mean), HA_SNL1 excluded.

Like rno-miR-449c-5p, for most of the 284 mature miRNAs the magnitude and direction of fold-change was similar in HA and LA rats (Figure 3). However, 3 miRNAs were regulated differentially (subtraction method, p < 0.05 after FDR correction). Use of log2 SNL/sham ratio as a metric of fold-change introduced a 4^th^ and randomization (‘bootstrap p-value’) brought the number to 5. The latter was nearly significant by FDR t-test (p = 0.069). Of these 5 *diff-reg* miRNAs (Table 1) only one, rno-miR-30d-5p, was abundantly expressed (RPM ~10,000, 15th ranked in abundance overall, Additional file 1: Table S1). This miRNA was down-regulated in HA and (slightly) up-regulated in LA. The others had RPM ranging from ~30-100 and are hence unlikely to be of major functional significance in the control of pain phenotype (see Discussion).

**Table 1:** Mature miRNAs that underwent significant differential up- or down-regulation following SNL nerve injury in HA vs. LA rats

**miRNA identifier name**	**Mean RPM (sham pools)**		**Mean RPM (SNL pools)**		***SNL-reg*** **subtraction method (SNL/sham fold-change)**		***diff-reg***	**qFDR**	**chr. #**
	**HA**	**LA**	**HA**	**LA**	**HA**	**LA**	**HA-LA**	**HA-LA**	
**subtraction method, all pools**									
**rno-miR-30d-5p**	**9,187**	**11,047**	**8,140**	**11,168**	**−0.121 (−1.129)**	**0.011 (1.011)**	**−0.132 (0.297)**	**<0.001**	7
rno-miR-378b	51.6	47.1	58.6	33.7	0.125 (1.136)	−0.331 (−1.398)	0.457 (0.533)	<0.001	5
rno-miR-322-3p	30.7	29.4	20.8	32.8	−0.384 (−1.476)	0.109 (1.116)	−0.494 (0.592)	<0.001	X
added by log2 ratio method or randomization *, all pools									
rno-miR-493-3p	79.8	66.3	87.9	91.0	0.138 (1.102)	0.455 (1.373)	0.317 (0.271)	<0.001	6
rno-miR-1839-5p *	112.1	108.9	100.3	129.1	−0.110 (−1.118)	0.168 (1.185)	−0.278 (.303)	<0.05	1
**added by HA_SNL1 pool exclusion**									
**rno-miR-125b-5p**	**3,758**	**2,537**	**2,718**	**2,398**	**−0.321 (−1.383)**	**−0.056 (−1.058)**	**−0.265 (0.325)**	**0.002**	**8**
**rno-miR-379-5p**	**201.9**	**187.5**	**188.6**	**231.6**	**−0.068 (−1.071)**	**0.210 1.235**	**−0.278 (0.306)**	**0.04**	6
rno-miR-369-3p	12.0	13.6	16.9	15.8	0.337 (1.408)	0.148 (1.162)	0.189 (0.246)	0.03	6
rno-let-7f-2-3p	11.7	8.1	10.4	12.8	−0.119 (−1.125)	0.448 (1.580)	−0.567 (0.705)	0.03	X
rno-miR-340-3p	8.9	11.5	12.6	9.3	0.346 (1.416)	−0.215 (−1.237)	0.561 (0.653)	0.03	10
rno-miR-6331	15.0	10.5	11.8	13.2	−0.241 (−1.271)	0.226 (1.257)	−0.467 (0.528)	0.05	6

Relatively abundant transcripts are in bold font.

### Pool consistency check and discretionary pool exclusion

**Figure 4:**
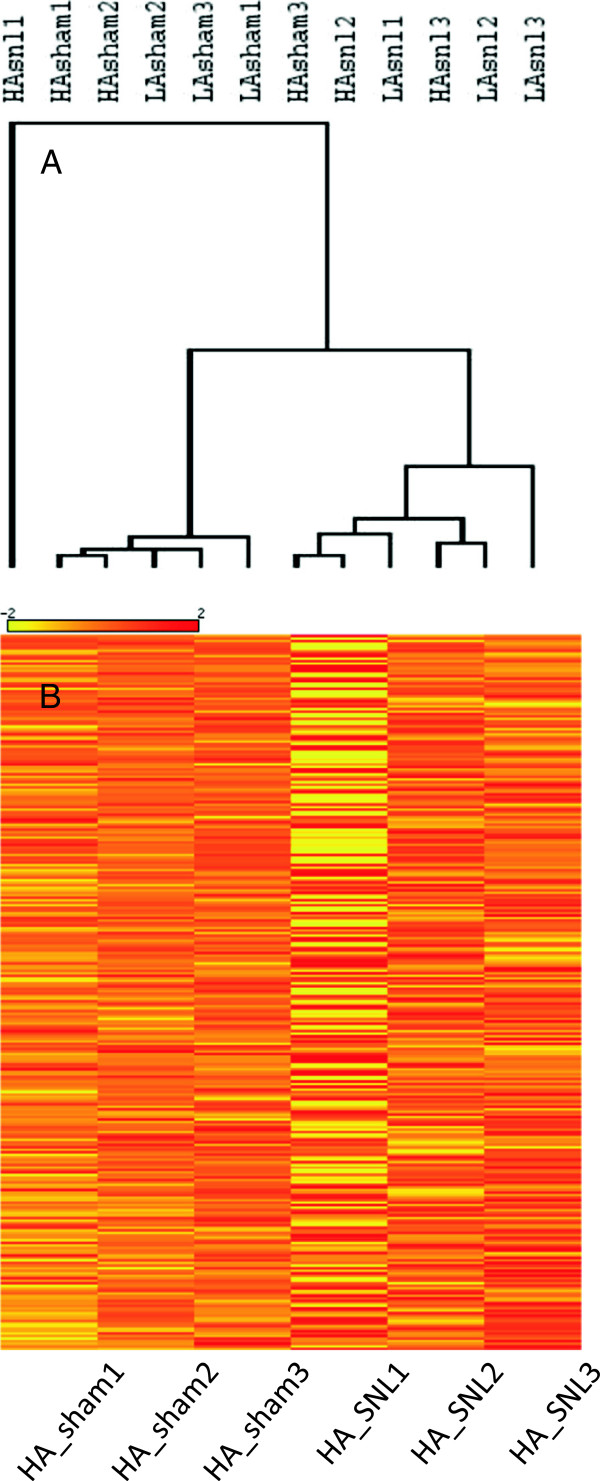
**Outlier status of the HA_SNL1 pool. A)** Hierarchical dendrogram based on pool similarity and **B)** HEAT maps [24] both illustrate the outlier status of the HA_SNL1 pool. HEAT map columns use color to represent the 284 miRNAs in the 3 HA_sham pools and the 3 HA_SNL pools. The miRNAs are arranged in decreasing order of average RPM in the HA_sham group. Color (scale above) represents relative RPM where 0 (orange) is the mean RPM of the 6 pools and +2 (red) and −2 (yellow) represent ±2 standard deviations [24].

Read counts were fairly consistent across pools. Considering all miRNAs in the 3 pools that made up each of the 4 experimental groups, the average coefficient of variation (CV = SD/mean) was only 0.20. However, if a biological or processing factor had increased variability in one of the pools, important signals could be obscured. To check on this we applied hierarchical cluster analysis to estimate pool consistency under the premise that the 3 pools within each experimental group ought to be more similar to one another than to pools in the other groups. Results revealed one HA_SNL pool, HA_SNL1, as a clear outlier (Figure 4A). Analysis of RPM values across miRNAs in the 6 HA pools also flagged HA_SNL1 as an outlier (Figure 4B). Finally, this conclusion was consistent with the fact that variability among pools in the HA_SNL group (CV =0.29) was much higher than in the other three groups (HA_sham = 0.14, LA_sham = 0.20, LA_SNL = 0.16). These observations justify excluding the outlier pool and basing results on only 11 of the 12 pools. However, to avoid potential appearances of bias we chose to present results obtained by computing *SNL-reg* and d*iff-reg* both with and without pool exclusion.

**Figure 5:**
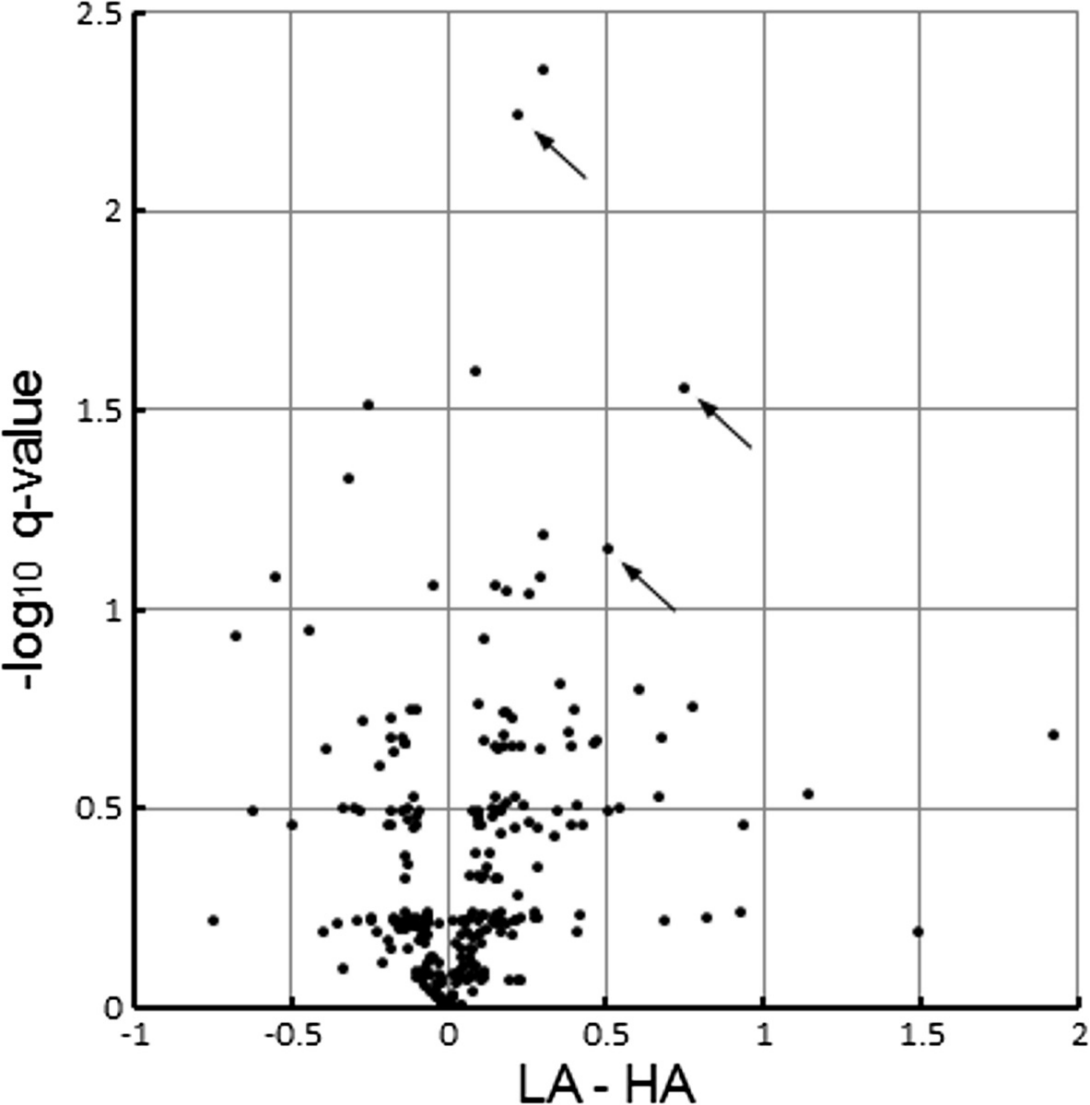
**Only a small fraction of miRNAs were regulated differentially by SNL nerve injury in HA compared to LA rats.** In this “volcano plot” each dot represents one of the 284 miRNAs detected in the DRGs studied, with the degree of differential regulation (*diff-reg*, LA-HA) plotted against the statistical significance of the diff-reg (FDR corrected q-values, given as -log_10_). LA-HA was calculated as the difference between the mean log2 ratios of LA_SNL/LA_sham and HA_SNLl/HA_sham (average of pool values). Data used for the plot excluded outlier pool HA_SNL1. miRs 125b-5p, 30d-5p and 379-5p are indicated with arrows. The other significantly diff-reg miRNAs were expressed at low abundance (Table 1).

None of the mature miRNAs (n = 284) or unique sequences (n = 3340) were eliminated because of the exclusion. However, reduced variability in the HA_SNL group following exclusion of the outlier pool yielded 6 additional significant differentially regulated miRs (Table 1). One, rno-miR-125b-5p, was abundantly expressed (RPM ~3000, ranked 30^th^ overall, Additional file 1: Table S1); it was down-regulated in HA and marginally so also in LA. An additional one was expressed at modest abundance (rno-miR-379-5p, RPM ~ 200) and the remaining 4 were rare (RPM < 15). Considering the two computations together, 11 miRNAs were differentially regulated at criterion significance after correction for multiple testing, 3 at abundant or moderate levels (Table 1, Figure 5). Additional file 1: Table S3 lists the top miRNAs ranked by the statistical significance of *diff-reg*. Additional file 1: Table S4 lists the top miRNAs ranked by the magnitude of *diff-reg*.

### PCR verification

**Figure 6:**
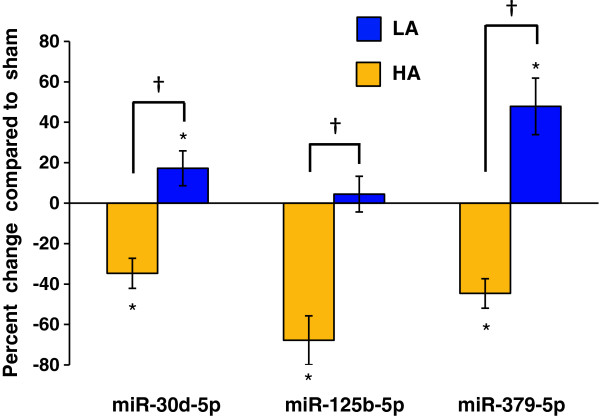
**Differential regulation of miRs 125b-5p, 30d-5p and 379-5p was confirmed using qRT-PCR.** Columns represent up- and down-regulation of miRNA expression in SNL operated HA and LA rats as a percent of sham control values. * p ≤ 0.05 vs. sham; † p ≤ 0.05 comparing regulation in HA vs. LA (*diff-reg*), one-way ANOVA followed by Turkey post-hoc test.

Regulation of the 3 abundant *diff-reg* miRNAs was verified using qRT-PCR. All were significantly downregulated in HA rats and 2 were significantly upregulated in LA rats. For all 3, significant differential regulation was confirmed (Figure 6). The additional 4 miRNAs checked also showed regulation consistent with observations using miRNA-seq.

### Up- and down-regulation of unique short RNA sequences

**Figure 7:**
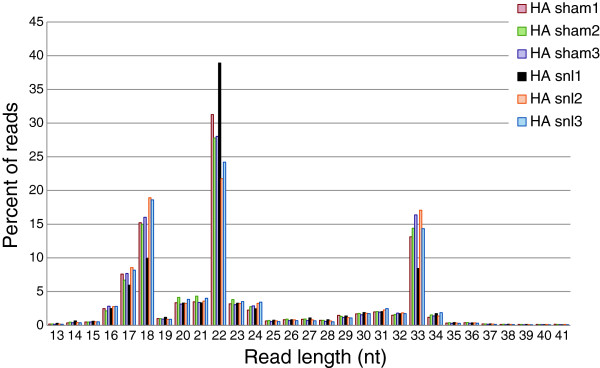
**Histogram showing that the small non-coding RNAs in the rat DRG fall into three categories by size: 17–18 nt, 21–23 nt (miRNAs) and 32–34 nt.** The HA_SNL1 pool (black columns) stands out as having relatively more miRNAs than the other pools.

Figure 7 shows a histogram of read lengths of all short RNA sequences in the 6 HA pools (results for LA were very similar). Note that the most common reads were ~22 nt in length, the miRNAs. However, there were two smaller peaks at 17–18 nt and 33 nt. Interestingly, in this analysis too, the HA_SNL1 pool emerged as an outlier, with an exceptionally high proportion of miRNAs and a low proportion of other sncRNAs. sncRNAs that are not miRNAs can regulate gene expression and the same may be true of individual miRNA sequences (isomiRs) among the many similar but non-identical ones that map to a particular canonical miRNA identifier.

To locate unique sequences that were regulated by SNL nerve injury we ran the *SNL-reg* and *diff-reg* computations using as input matricies the 3340 reads-level sequences detected at criterion abundance in the experimental pools. No individual unique sequences were significantly regulated by SNL nerve injury after FDR correction (in all pools, or after HA_SNL1 exclusion). However, 7 showed significant differential regulation (all pools analysis). It is unlikely that these hits were sequencing artifacts as each occurred in all 12 pools, with RPM ≥10 in most. The 7 included one isomiR (of rno-miR-101a-3p, RPM ~ 10), two rRNA fragments, two tRNA fragments and two unidentified. Analysis after excluding the HA_SNL1 pool yielded an additional 33 *diff-reg* sequences. Among these were one isomiR of rno-miR-130a-3p and one of rno-miR-125b-5p (a miRNA already identified as differentially regulated, Table 1). Interestingly, the canonical miRNAs of all three *diff-reg* rat isomiRs were abundantly expressed (RPMs ~3,500, 450, 3,150 respectively). The differentially regulated isomiR of rno-miR-125b-5p (RPM ~35) differed from the consensus sequence of its canonical miRNA only by deletion of the nucleotides **uga** from the 3’ end [ucccugagacccuaacuug**uga**]. It is noteworthy that individual isoforms of a given miRNA may be regulated to different degrees by nerve injury than others, and than the miRNA’s consensus sequence. As such, some might be more potent regulators of gene expression than the other isomiRs that comprise the miRNA.

The remaining 31 unique sequences included one isomiR of a miRNA previously recognized in the Tasmanian devil but not in the rat (sha-miR-5105), 9 rRNA fragments, 13 tRNA fragments and 8 unidentified sequences. All but 3 of the 33 sequences (including the isomiRs) had RPM < 50. The exceptions were one rRNA (RPM ~ 800) and two tRNA fragments (RPM ~ 700 and 100; Table 2). We further explored their alignment patterns and found that all 3 RNA elements are mapped by >2000 unique reads. These reads were not homogenously spread over the sequences, but were strongly concentrated to certain regions. This suggests that the fragments were products of regulated processing rather than random RNA degradation.

**Table 2:** Unique sequences of miRNA isoforms (isomiRs) and other sncRNAs (with RPM ≥100) that underwent significant (qFDR < 0.05) differential up- or down-regulation following SNL nerve injury in HA vs. LA rats

**Small RNA type and sequence**	**Mean RPM**		**Mean RPM**		***SNL-reg***		***diff-reg***
(canonical miRNA or RNA type)	(sham pools)		(SNL pools)		subtraction method (SNL/sham fold-change**)**		
	HA	LA	HA	LA	HA	LA	HA-LA
unique-sequence miRNA isoform (isomiR), all pools, and those added by HA_SNL1 pool exclusion *							
UAUAGUACUGUGAUAACUGACU (rno-miR-101a-3p)	11.7	9.3	9.9	11.3	−0.167 (−1.182)	0.194 (1.215)	−0.361 (0.397)
CAGUGCAAUGUUAAAAGGGC (rno-miR-130a-3p) *	38.0	41.9	48.2	45.0	0.237 (1.268)	0.071 (1.074)	0.166 (0.194)
UCCCUGAGACCCUAACUUG (rno-miR-125b-5p) *	47.8	30.4	35.5	33.1	−0.295 (−1.346)	0.085 (1.089)	−0.380 (0.435)
UGGACGGUGUGAGGCC (sha-miR- 5105) *	11.0	15.6	13.5	11.9	0.204 (1.227)	−0.269 (−1.311)	0.473 (0.537)
**unique-sequence sncRNAs with RPM ≥100, after HA_SNL1 pool exclusion**							
AGCCGCCUGGAUACCGCAGCUAGGAAUAA (rRNA)	1843.3	616.5	548.0	666.0	−1.846 (−3.363)	0.078 (1.080)	−1.924 (2.443)
GCAUUAGUGGUUCAGUGG (tRNA)	621.1	719.9	771.1	713.5	0.215 (1.242)	−0.009 (−1.009)	0.224 (0.251)
UCAUUGGUGGUUCAGUGG (tRNA)	88.6	111.5	114.1	103.4	0.252 (1.288)	−0.075 (−1.078)	0.327 (0.366)

### Novel rat miRNAs

We detected 13 pre-miRNAs novel for the rat. For 5 of the pre-miRNAs we detected mature miRNAs from both 3’ and 5’ arms, for a total of 18 novel mature miRNAs (Additional file 1: Table S5). Among the 18, 14 had homologs in other species (most in the mouse) and 4 are reported here for the first time. We have named most of the 14 rat homologs using the mouse identifier number prefixed by rnoH (rnoH-miR). One of the 14 was previously reported in a variety of mammalian species (several primates, cow, dog and horse), but not in rodents. The best match was the horse homolog eca-miR-1271, and hence our designation rnoH-miR-1271. One of the 18 was recognized in the newly released miRBase-v20 (rno-miR-155-5p). For one rat pre-miRNA both mature miRNAs were expressed and their sequences matched those in the mouse perfectly (mmu-miR-486; the complete pre-miRNA sequence is unknown for the rat). The -5p sequence was highly abundant in rat DRGs (RPM ~23,500) ranked 7^th^ in Additional file 1: Table S1 and S5). The 4 sequences (2 pre-miRNAs) with no known homologs in miRBase-v19 (or -v20) were designated rno-miR-X1 and X2 in Additional file 1: Table S5. All occurred at very low abundance, but were present in many pools and hence are unlikely to represent read errors. Four of the 18 novel miRNAs were among the 50 most highly expressed miRNAs in the rat DRG (mmu-miR-486-5p, rnoH-miR-148a-3p, rnoH-let-7 g and rnoH-miR-676-3p, set in italics in Additional file 1: Table S1). None of the novel miRNAs showed significant *SNL-reg* or *diff reg.*

### Bioinformatic evaluation of the differentially regulated miRNAs

#### Validated targets

We searched for validated target mRNAs for the 11 significantly *diff-reg* mature miRNAs and for the canonical miRNAs for which one or more unique sequence isomiRs was differentially regulated (although not the canonical miRNA itself; Tables 1 and 2). In addition, we queried the two miRNAs that were differentially expressed at baseline (HA_sham vs. LA_sham). Results are summarized in Table 3. A total of 53 validated target genes were identified for the 11 miRNAs. Most had no known validated targets, but the two most abundant ones (rno-miR-30d and rno-miR-125b-5p) had 50 amongst them. Three targets stood out as known players in pain physiology. These were Tnf, Bdnf and Stat3, genes that code for the cytokine TNF (tumor necrosis factor [25-27], the growth factor BDNF (brain-derived neurotrophic factor [28-30]; and the transcription factor STAT3 (signal transducer and activator of transcription 3 [31,32]). mRNA for TAC1 (tachykinin precursor 1) is a target of the canonical miRNA of one of the *diff-reg* isomiRs (Table 3).

**Table 3:** Number of validated mRNA targets of miRNAs that were differentially up- or down-regulated by SNL nerve injury

**miRNA identifier**	**RPM mean HA + LA (sham)**	***diff-reg *** **abs. value, by subtraction**	**No. of verified mRNA targets**	**Target genes relevant to pain physiology**
**Differentially regulated miRNA**				
rno-miR-378b	49.4	0.457	0	
rno-miR-322-3p	30.1	0.494	2	-
**rno-miR-30d-5p**	**10,117**	**0.132**	**5**	**Bdnf**
rno-miR-493-3p	73.1	0.316	0	
rno-miR-1839-5p	110.9	0,278	0	
**rno-miR-125b-5p**	**3147.6**	**0.265**	**45**	**Tnf, Stat3**
rno-miR-369-3p	12.8	0.189	0	
rno-let-7f-2-3p	9.9	0.567	0	
rno-miR-340-3p	10.2	0.561	1	-
rno-miR-6331	12.8	0.467	0	
rno-miR-379-5p	194.7	0.278	0	
**Canonical miRNA of unique sequence isomiR (*diff-reg *** **of isomiR)**				
rno-miR-101a-3p	1735.9	0.079	28	-
rno-miR-130a-3p	470.8	0.160	16	**Tac1**
**Differential expression at baseline (HA_sham vs. LA_sham) all pools**				
rno-miR-184	25.2	0.550	4	-
rno-miR-6325	16.4	0.114	0	

#### Predicted targets

**Figure 8:**
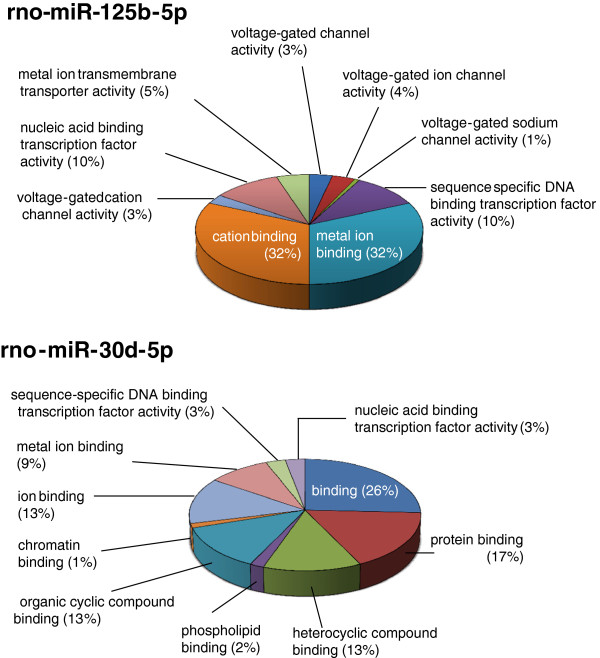
Pie charts show the Gene Ontology (GO) molecular function terms that are enriched compared to the overall genome, among the mRNA targets predicted by the TargetScan algorithm for miR-125b-5p (n = 451) and miR-30d-5p (n = 1094).

For the 3 *diff-reg* miRNAs of high or moderate abundance we also explored predicted, but not (yet) verified, mRNA targets. Since hundreds were found for each, we carried out a systems level analysis to define which Gene Ontology (GO) molecular function terms were enriched that characterize the collection of target genes in comparison with the genome in general. The most notable observations were enhanced ion, protein and nucleic acid binding, and hence possibly regulation, among the protein targets of rno-miR-30d-5p and rno-miR-125b-5p, and enriched ion channel activity, including Na^+^ channel activity, in rno-miR-125b-5p (Figure 8).

## Discussion

We applied a distinctive genome-wide search strategy to rat selection-lines that have similar genetic background, but contrasting pain phenotype. The aim was to discover miRNAs related to neuropathic pain while avoiding processes such as cell metabolism, regeneration and apoptosis that are also triggered by nerve injury but that do not differ between the lines [33]. Overall we encountered only three miRNAs that were present in significant copy number and were regulated differentially by nerve injury in HA vs. LA rats. These were rno-miR-30d-5p and rno-miR-125b-5p which were expressed abundantly and rno-miR-379-5p which was expressed at modest copy number. Eight additional *diff-reg* miRNAs were found. But since they were rare, it is unlikely a *priori* that they are functionally important. This determination rests on the observation that most miRNAs for which a significant biological role has already been established are abundantly expressed. Furthermore, experimental use of decoy libraries has shown that in general, miRNAs expressed at 100 RPM or less are unlikely (<2%) to detectably suppress the expression of any target mRNA [34], let alone to have a prominent behavioral effect. Indeed, the 8 low-abundance miRNAs might not have been differentially regulated at all. Some or all of these hits probably met the significance criterion by chance (type 1 error). Since the ~3.5 × 10^6^ miRNA reads in each pool represent only ~0.001% of the actual number of miRNA molecules loaded into the sequencer, stochastic effects can generate false positives, especially for low-abundance sequences [35]. Consider that a random (or read-error) difference of only a few reads on a baseline RPM = 10 translates to a much larger fold-change than the same error on a baseline of RPM = 1000.

Genome-wide scans typically yield large numbers of hits requiring prioritization based on factors such as magnitude of fold-change ratio or occurrence in several different pain models [36]. This is risky and possibly even counterproductive as it might highlight processes such as regeneration and metabolism that are generic to nerve injury but not necessarily to pain. The approach is also likely to obscure transcripts related to particular pain conditions but not others. Our approach sidesteps the need for secondary screens for obtaining a short-list. Indeed, we cast a broad net which included *diff-reg* miRNAs obtained with and without exclusion of outlier pool HA_SNL1, two different methods of calculating regulation and canonical miRNAs of unique sequence *diff-reg* isomiRs. Nonetheless, a very small number of *diff-reg* miRNAs were found and these turned out to be regulators of established major pro-nociceptive molecules. This affirms the intrinsic power of the strategy to prioritize pain-relevant transcripts [33].

We believe that the key factor was comparison of animals with very similar pedigree but strongly contrasting pain phenotype. Standard commercial strains show robust differences in constitutive miRNA expression [37] while in HA/LA rats we found only two miRNAs with a constitutive expression difference at baseline (sham-operated). Even these are questionable given their very low abundance (RPM ~ 10). Another factor was harvesting DRGs only 3 days postoperatively. Up- or down-regulation of miRNAs after nerve injury is progressively more robust over the first weeks postoperative. By 2–4 weeks a much larger fraction of expressed miRNAs are regulated [15,38,39]. Since differential pain phenotype in our rats is robust by 3 dpo [3] sequences that begin to show regulation only later are unlikely to be causative. We conclude that rno-miR-30d-5p, rno-miR-125b-5p, and perhaps rno-miR-379-5p are fundamental to the contrasting neuropathic pain phenotype in HA vs. LA rats. Since the contrast includes spontaneous pain as well as tactile allodynia [3] these miRNAs may be important players in neuropathic pain in general. Regulation of these miRNAs in the DRG following nerve injury has been noted previously (e.g. [38]), but they have not been specifically implicated as key factors in neuropathic pain.

### Downstream targets of the differentially regulated miRNAs

The 8 rare *diff-reg* miRNAs, and the modestly expressed rno-miR-379-5p, had only 3 verified mRNA targets among them, none that hinted at a role in pain physiology. The two abundantly expressed *diff-reg* miRNAs had more, including 3 that code for well-known pro-nociceptive compounds, TNF, BDNF and STAT3 (Table 3). This represents notable enrichment of pain-related molecules. Since both miRNAs were more down-regulated in pain prone HA rats than in LA rats, and miRNAs suppress the expression of their mRNA targets, this regulation is expected to *increase* target gene product levels in HA vs. LA. Indeed, TNF and BDNF are upregulated and released by DRG neurons and glia in inflammatory and neuropathic pain models and are strongly pro-nociceptive [25,26,28,29,40,41].

Interestingly, specific knockout of BDNF expression in small diameter neurons was found to have little effect on pain behavior 3 days postoperatively, indicating that BDNF in nociceptors does not play a significant role in neuropathic pain phenotype [42]. However, BDNF expression in larger DRG neurons (Aβ-afferents) and glia is not eliminated in these knockout mice. Indeed, as noted, BDNF expression is markedly increased in large diameter afferents following axotomy [28,29]. This reiterates the special role of activity in Aβ afferents as drivers of neuropathic pain [43]. In addition, it suggests that regulation by miRNAs might be a particularly important factor in the phenotypic switching of Aβ touch-signaling neurons, a change that contributes importantly to their seemingly paradoxical involvement in pain perception in the event of neuropathy [5,44].

STAT3, a transcription factor in the JAK-STAT pathway, is activated in sensory neurons and glia following noxious stimulation and nerve injury and is also associated with potentiation of pain [31,45]. Thus, the selective downregulation of all 3 *diff-reg* miRNAs likely contributes to the prominent afferent hyperexcitabilty of HA rats [4] and hence to their marked neuropathic pain phenotype.

As noted above, Tnf, Bdnf and Stat3 have already been verified experimentally as targets of rno-miR-30d-5p and rno-miR-125b-5p, and functional assays involving primary afferents have already established all three as major players in pain physiology. Additional corroboration has therefore not been undertaken here, although the issue of whether miRNA-regulated expression is selective to particular types of DRG neurons, but not others, deserves follow-up. We conclude that neuropathy-induced regulation of these *diff-reg* miRNAs contributes to the control of individual variability in neuropathic pain phenotype. Further research is required to determine whether some of the other known or predicted gene targets of rno-miR-30d-5p and rno-miR-125b-5p might also contribute. In this regard it is noteworthy these targets in part relate to inflammatory processes and not strictly to processes of neuropathic (electrical) excitability. Indeed, it is clear that inflammatory mediators exacerbate ectopia in afferent neurons, blurring the line between neuropathic and inflammatory pain [1,15,26,29,41].

A key strength of whole-genome search strategies is their ability to reveal elements not previously associated with pain processing. TNF, BDNF and STAT3 were highlighted because of prior knowledge of their prominent role in pain. However, *in vivo* all of the genes targeted by our 3 miRNA hits are potentially down-regulated. We must imagine that the contrasting pain phenotype of HA vs. LA rats is orchestrated by differential regulation of the entire constellation of their target genes. The list of targets already validated, and those yet to be validated, constitutes a useful pool of pain-related genes. In this regard it should be noted that since RNA was extracted from whole DRGs, non-neural cells in the ganglia might also have contributed to the yield of *diff-reg* miRNAs.

### Unique sequence miRNA isoforms (isomiRs) and other sncRNAs

Small sequence differences among isomiRs can affect binding affinity to mRNA targets [35,46]. Thus, a particularly potent individual isomiR, or a small subset, could be responsible for a large proportion of the functional effect of the canonical miRNA. Indeed, in three instances the fold-change of one isoform reached statistical significance even though that of its canonical miRNA did not (Table 2). In each case the isomiR contributed only a small fraction of the overall RPM of the miRNA. The canonical miRNA of two of these, rno-101a-3p and rno-miR-130a-3p, had a variety of validated mRNA targets (n = 44, Table 3). Differentially regulated isomiRs merit further exploration. Likewise, *diff-reg* sncRNAs that are not miRNAs might be significant mediators of pain variability. Our reads level analysis identified a number of candidates. Many of these were parsed as tRNAs or rRNAs. Three of them were relatively abundant (Table 2) and showed signs of being processed rather reflecting random RNA degradation. These sequences also merit further study.

### The contribution of miRNA regulation to neuropathic pain

Nerve injury is consistent in causing negative symptoms, hypesthesia and numbness, in the corresponding body part. Positive symptoms, paresthesias and pain, are much more variable indicating that the nerve lesion is not causative in itself. Among major limb amputees, for example, <25% report persistent severe phantom pain [47] while <0.01% of adults have trigeminal neuralgia among the ~17% who have the (putative) causative nerve lesion [48]. Genetic polymorphism is believed to be a key co-factor determining whether or not nerve injury will lead to neuronal hyperexcitability and neuropathic pain. However, the widely used approach of simply identifying genes regulated by nerve injury is an inefficient route for discovering the polymorphisms and pathways that play a special role in pain processing. The problem is that thousands of mRNAs are regulated following nerve injury [21-23,33]. It is very difficult to delineate which contribute to enhanced pain sensibility and which are related to other processes triggered by nerve injury. A systematic means is required to solve this problem. The strategy used here, examining differential effects of nerve injury in animals that are genetically similar but different in pain phenotype, represents a way forward. Remarkably few differentially regulated miRNAs and other sncRNAs were identified, and even fewer remained candidates after (tentative) exclusion on the basis of low copy number. Prioritizing candidate genetic elements using this strategy is likely to have general applicability to problems in which there is a variable link between the precipitating pathology and the resulting phenotype.

## Methods

### Animals and surgery

Observations were made on DRG tissue taken from adult HA and LA rats of both sexes raised in our Institute animal facility under SPF (specific pathogen free) conditions. HA and LA rats were derived from outbred Wistar-based Sabra strain rats by genetic selection as described by Devor and Raber [2]. They show contrasting pain phenotype in both the neuroma and the spinal nerve ligation (SNL) models of neuropathic pain [1,3,49]. The animals used for this study were from the 63rd (HA S63) and the 61st (LA S61) generations of selection and had been inbred for the previous 12 generations. The animals were contemporaneous despite the different generational designations. This is due to somewhat quicker selection in the HA line. Before and after surgery animals were housed in plastic cages at an ambient temperature of 22 ± 1°C and a 12 hr diurnal light cycle with lights on at 6:00. Pelleted food (Teklad, #2018FC + F) and water were provided *ad libitum*. Cages were cleaned twice weekly. Experiments were approved by the Institutional Animal Care and Use Committee (IACUC) of the Hebrew University of Jerusalem, and followed IASP (International Association for the Study of Pain) guidelines for the humane use of laboratory animals.

Four equally sized experimental groups were formed from HA and LA rats that underwent either SNL or sham surgery. They are designated HA_SNL, HA_sham, LA_SNL, and LA_sham. Rats were deeply anesthetized with 4% chloral hydrate (400 mg/100 g body weight, i.p.). Using antiseptic precautions the lower lumbar spine was exposed and the L6 transverse process was removed, bilaterally in most animals, to reveal the left L4 and L5 spinal nerves. These were transected 4–5 mm distal to the DRG (no ligature). A carbon mark was placed near the cut nerve ends to facilitate postmortem identification. In sham-operated rats, the spinal nerves were exposed, but not touched. The incision was then closed in layers using discontinuous silk sutures in muscle and Michel skin clips. Antibiotic powder was applied to the incision and a single prophylactic injection of ampicillin was given (50 ku/kg i.m.; Sandoz, Kundl, Austria). The accuracy and completeness of the intended surgery was confirmed visually at the time of DRG harvesting. This included postmortem dissection of the lumbosacral plexus in a sample of rats.

### Tissue collection, RNA extraction and sequencing

Three days after SNL or sham surgery the animals were again anesthetized with chloral hydrate and the L4 and L5 DRGs were removed and trimmed of fat and connective tissue. The spinal nerve and roots were then shortened to about 1 mm, and the DRGs were rinsed in sterile saline, immersed in ice cold RNA stabilization reagent (RNAlater, 150 μl, Qiagen GmbH, Hilden, Germany, product # 76101) and rapidly frozen. The rats were then killed by anesthetic overdose and cervical dislocation. Only ~2 min elapsed between DRG removal and freezing. L4 and L5 DRGs from the operated side of each rat were collected in individual labeled Eppendorf vials and stored at −70°C pending RNA extraction. Each vial contained one DRG. To ensure a high enough starting RNA concentration we formed biological samples by pooling 12 DRGs. These were provided by 3 or 4 rats from each of the four experimental groups, balanced for sex but otherwise chosen at random. Thus, for each group there were three biological replicates (pools) for a total of 12 pools (and 144 DRGs) altogether. The pools were designated HA_SNL1, 2 and 3, LA_sham1, 2, 3, and so forth.

Total RNA was isolated using the mirVana™ miRNA Isolation Kit (Applied Biosystems, Carlsbad, CA, AM #1561) following manufacturer instructions. The total miRNA fraction was dissolved in nuclease-free water and purification steps were performed using Qiagen kit #79254 following manufacturer instructions. RNA concentration was determined using the NanoDrop spectrophotometer (NanoDrop Technologies, Wilmington, DE) and the quality of the total RNA was checked by gel analysis using the total RNA Nanochip assay on an Agilent 2100 Bioanalyzer (Agilent Technologies GmbH, Waldbronn, Germany). 250 ng of RNA obtained from each pool of 12 DRGs was used as starting material for miRNA deep-sequencing analysis. Sequencing was carried out at GATC Biotech (Constanz, Germany) using the Illumina Hiseq 2000 sequencer, following established in-house protocols [GATC Biotech: DNA Sequencing and Bioinformatics **(**http://www.gatc-biotech.com/en/index.html).

For each of the 12 pools GATC Biotech provided raw Fastq files that listed the number of reads of each unique RNA sequence with length ranging up to 41 nucleotides (≤41 nt). After appropriate editing and mapping (below) these files were used to identify miRNAs present in the rat DRGs, to determine effects of SNL nerve injury (up- and down-“regulation” (*SNL-reg*)) and to identify miRNAs for which nerve injury-induced regulation differs between the two rat lines (“differential regulation” (*diff-reg*)).

### Sample validation by PCR

RNA-seq data were validated with quantitative real-time polymerase chain reactions (qRT-PCR) in a sample of 7 miRNAs including the 3 most abundant ones with significant *diff-reg* (see Results). To generate miRNA-specific first strand cDNA 20 ng of total RNA was reverse transcribed with miRNA-specific RT primers using the TaqMan® MicroRNA Reverse Transcription Kit (Applied Biosystems, #4366597) following the manufacturer’s instructions. Prepared cDNA template was PCR amplified in each reaction using corresponding miRNA-specific primers and TaqMan® Universal Master Mix II - no UNG (Applied Biosystems, #4440040, manufacturer’s instructions) on a LightCycler® 480 Real-Time PCR System (Roche Diagnostics GmbH, Germany). The target miRNAs (and corresponding assay numbers) were: rno-miR-23a (000399), rno-miR-26b (000407), rno-miR-30-5p (000420), rno-miR-101b (002531), rno-miR-125b-5p (000449), rno-miR-379 (001138) and rno-miR-431 (001979). Expression levels were normalized to that of U87, a small nucleolar RNA (assay number: 001712).

### Editing and mapping

#### Editing

Our starting point was the Fastq file for each of the 12 pools. Using miRanalyzer (standalone version [50]), the raw reads in each pool were sorted into unique sequences and their corresponding abundances (read counts). These were edited to construct final expression matrices suitable for calculating nerve injury-induced regulation (*SNL-reg*) and differential regulation (*diff-reg*). First, adapter sequences were trimmed from the raw reads by forcing the detection of at least 10 nt of the adapter, allowing one mismatch. Reads for which no adapter sequence was detected, and reads shorter than 16 nt, were removed from the analysis retaining only sequences 16–41 nt in length. Note that the size filters retained RNAs 16–20 nt and 24–41 nt in length, forms too small and large to be miRNAs (Figure 7). The rationale was that some sncRNAs in these size ranges might nonetheless play a regulatory role analogous to miRNAs [15,51,52]. The outcome of editing was an expression matrix listing the total number of reads of each unique small RNA sequence (“read-level counts”).

#### Mapping

The edited reads were then assigned a type (annotated) by mapping to several sequence libraries, in a fixed order. Our mapping pipeline took into account different ways in which RNA molecules can be post-transcriptionally modified. For example, in addition to RNA editing it has been shown recently that RNA molecules may be modified by adding uracils (uridylation) and adenines (adenylation) to the 3’ end (NTA, non-templated addition, [53]). Since these additions are not present in the library consensus sequences, NTAs can produce mismatches in the alignment between the read sequence and the miRNA consensus sequence. Strongly modified reads might not be mapped at all. To correct for this we used Bowtie seed alignment [54]. Briefly, we aligned only the first 20 nt of each read (the “seed”) to the sequence libraries, ignoring all mismatches beyond the 20th nt. In this way, NTAs do not cause mismatches and the corresponding sequences are mapped. Finally, to accommodate for sequencing errors and rare RNA editing events we allowed one mismatch when mapping to known miRNAs, and two mismatches when searching for putatively homologous miRNAs.

Note that miRNAs with unique miR identifier names in the miRBase library [55-58] usually map more than one unique read sequence. To refer to the set of unique sequence isoforms that map to a particular miR identifier (sometimes dozens) we use the term “isomiRs”. The isomiRs of a particular miRNA are thought to derive from a unique DNA level miRNA gene, but are non-identical due to post-transcriptional processing which deletes or adds one or two nt’s [35,46]. Mapping used the following seven libraries, in this order: (i) known rat (Rattus norvegicus) miRNAs (rno-miRs, miRBase-v19 [58], (ii) all known miRNAs (species-miR, miRBase-v19), (iii) REfSeq genes [59] (iv) Rfam (v. 11) [60] (v) t-RNAs [61] (vi) RepBase repetitive DNA [62] and (vii) piwi-RNAs, obtained using the NCBI nucleotide database using “piRNA, piR, rattus norvegicus” as search terms. Our main analysis used the first two libraries. The others were used for annotating reads-level results. Once a read was successfully aligned to a named consensus sequence in a library, the now identified read sequence was removed from the input to the next library in the order. This prevented mapped sequences from being erroneously counted twice. Sequences that ran through all libraries without being assigned a miRNA identifier name or being otherwise annotated were left unnamed, but were nonetheless included in the final reads-level matrix used for calculating SNL-regulation and differential regulation.

Following mapping, we converted the read count of each short RNA element into “reads per million” (RPM). This was done independently for each of the 12 pools in two alternative ways: 1) In “within-library normalization” the number of reads assigned to a unique identifier name in a given library (e.g. rno-miR-143-3p in library (i)) was normalized to the total number of reads identified by all identifiers in that library. 2) Alternatively, in “reads-level normalization” the number of reads of each unique sequence in a pool was normalized to the total number of reads of all sequences in that pool. Finally, we excluded low abundance miRNAs by deleting all RNA elements (miRNAs, other sncRNAs and un-named sequences) for which RPM was not ≥10 in at least 3 of the 12 pools.

#### Test for pool consistency

The 12 DRGs in each pool originated from 3–4 rats and had been individually hand dissected and trimmed. To evaluate the possibility of an abnormality in one or more of the pools we checked for internal consistency under the assumption that pools compared within each experimental group should be more self-similar than pools compared across groups. This was done using a hierarchical clustering algorithm (average linkage) implemented with Cluster 3.0 [63]. The distance metric used was the pairwise correlation coefficient between RPM profiles of two samples.

#### Calculation of miRNA regulation and differential regulation

We calculated for each of the 12 pools *SNL-reg*, the degree to which the expression of individual named miRNAs and other small RNA sequences was up- or down-regulated by SNL nerve injury. Then we identified elements for which nerve injury-induced regulation *differed* significantly, in degree and/or direction, in HA vs. LA rats (*diff-reg*). These calculations were based on RPM values of the RNA element, and were done separately on two different data matrixes: a) Mature miRNAs and b) All unique read sequences, including individual miRNA isoforms (isomiRs) and other sncRNAs.

#### SNL injury-induced regulation (SNL-reg)

Because there is no unique measure of regulation we calculated SNL-reg in two ways. The first was (SNL-sham)/(mean of SNL + sham). Thus, for each mature miRNA the mean RPM of the 3 HA_SNL pools was subtracted from the mean RPM of the 3 HA sham pools and the result was divided by the average of all 6 HA pools. This was repeated for LA. Nerve injury-induced up-regulation yields positive values, down-regulation negative values and no change zero. We also calculated fold-change ratio. Since SNL/sham yields values >1 for up-regulation, but positive fractional values (rather than negative values) for down-regulation, log2 of SNL/sham was used for statistical computations. The null hypothesis of no difference between SNL and sham was tested using 1-tailed t-tests. The FDR limit [64] was also computed (for α = 0.05) and used to correct for multiple testing as explained below. For the much larger reads-level matrices of unique sequences *SNL-reg* was calculated using the DESeq routine in the R statistical software package [65].

#### Differential regulation (diff-reg)

*Diff-reg* for mature miRNAs was evaluated statistically as follows: For each element in each of the 3 HA_SNL pools we calculated *SNL-reg* with respect to *each* of the HA_sham1, HA_sham2 and HA_sham3 pools. Thus, we calculated (HA_SNL1- HA_sham1)/mean, (HA_SNL1- HA_sham2)/mean, (HA_SNL1- HA_sham3)/mean, (HA_SNL2- HA_sham1)/mean and so forth. This was repeated for LA. The result was 9 estimates of *SNL-reg* for HA and 9 estimates for LA, each with a corresponding mean ± SD (standard deviation). The same was done using log2 ratio fold-change. For unique sequences only log2 ratio was used. To obtain *diff-reg*, we compared mean *SNL-reg* from the HA and LA lines using 2-tailed t-tests followed by computation of the corresponding FDR. For extra confidence we also implemented an algorithm that compared RPM means in 10,000,000 random runs for each miRNA [66]. This approach is free of assumptions concerning the particular distribution of the underlying RPM values.

#### Statistical correction for multiple testing

For both *SNL-reg* and *diff-reg* the null hypothesis of no regulation was rejected for all RNA elements for which uncorrected p ≤ FDR limit [64]. DESeq also corrects for multiple testing, using the Bonferroni method (padj). Corrected p ≤ 0.05 was considered significant. Mean values are given throughout ± SD.

### Bioinformatic data analysis

#### Prediction of novel miRNAs

We first pooled all 12 samples together. After adapter trimming and collapsing the redundant sequences into unique read counts, we removed all sequences with fewer than 5 reads. The remaining reads were then used to predict novel miRNAs. Using miRanalyzer [50], we first clustered reads that mapped to the same position on the chromosome, allowing a window of 2 nt around the start position of the most frequent read. Several putative pre-miRNAs were then extracted from the genome sequence around each read cluster. These sequences were analyzed for “candidates” by RNAfold retrieving of those with the lowest energy that show a hairpin secondary structure. Finally, a machine learning algorithm was applied to each candidate yielding the final novel sequence predictions. After obtaining the predicted miRNAs we applied further thresholding, as follows, in order to reduce the number of false positive predictions: i) minimum read count ≥ 10 for the most frequent read, ii) length of the most frequent read between 20 nt and 23 nt, and iii) fluctuation around the 5’ start of the mature miRNA within 2 nt. Last, for rat specific novel miRNAs, i.e. those not previously recognized in any species, we required the existence of both arms in the sequencing data to show perfect 2 nt 3’ overhangs as produced by Drosha/Dicer.

#### Validated and predicted gene targets

Genes that have been experimentally validated as functional targets of the *diff-reg* miRNAs identified were retrieved from the miRTarBase repository [67]. These were reviewed by the authors to tag the ones salient to pain physiology. For the 3 most abundantly expressed *diff-reg* miRNAs identified we also searched TartgetScan [68] for gene targets that are predicted based on their sequences, but not (yet) documented experimentally, to be plausible functional targets. These were further scanned for Gene Ontology molecular function enrichment terms by uploading the targets of each of the 3 miRNAs independently to WebGestalt (WEB-based GEne SeT AnaLysis Toolkit) following all default parameters for the GO analysis [69,70].

## Competing interests

The authors declare that they have no competing interests with respect to this paper.

## Authors’ contributions

KKB carried out the *in vitro* experiment and much of the data analysis, MH also contributed importantly to the data analysis, AL carried out the *in vivo* procedures, RK and MD conceived of and financed the project and MD drafted the manuscript with feedback from all of the other authors. All authors read and approved the final manuscript.

## Supplementary Material

Additional file 1: Table S1 Top 50 miRNAs ranked in order of abundance in the L4 and 5 DRGs of sham operated rats. **Table S2.** miRNAs nominally up- and down-regulated (p<0.05 before correction for multiple testing) after SNL nerve injury in HA (n=23) and LA rats (n=35; ranked by p-value). *SNL-reg* was calculated from RPM by the subtraction method: (SNL-sham)/(SNL+sham). None showed significant *SNL-reg* after FDR correction for multiple testing. **Table S3.** Top miRNAs ranked by nominal (uncorrected) statistical significance of differential regulation between HA and LA DRGs following SNL nerve injury (subtraction method, HA_SNL1 pool excluded). miRNAs with significant *diff-reg* after FDR correction (Table 1) are set in italics. **Table S4.** Top miRNAs ranked by magnitude of differential regulation between HA and LA DRGs following SNL nerve injury (subtraction method). **Table S5.** miRNAs identified in rat DRGs that have not been previous reported in the rat (miRBase-v19). The ones with the prefix rnoH have homologs of the same number in other species (mostly the mouse). One was listed in the rat miRBase-v20. The ones numbered X1, X2 do not have homologs in any other species (sequence noted). Click here for file
