# Supplementary material for: Sources of individual variability: miRNAs that predispose to neuropathic pain identified using genome-wide sequencing
Source: Mol Pain. 2014 Mar 19;10:22. doi: 10.1186/1744-8069-10-22 (PMC4113183; doi:10.1186/1744-8069-10-22)
Supplement: Additional file 1: Table S1 — Top 50 miRNAs ranked in order of abundance in the L4 and 5 DRGs of sham operated rats. Table S2. miRNAs nominally up- and down-regulated (p<0.05 before correction for multiple testing) after SNL nerve injury in HA (n=23) and LA rats (n=35; ranked by p-value). SNL-reg was calculated from RPM by the subtraction method: (SNL-sham)/(SNL+sham). None showed significant SNL-reg after FDR correction for multiple testing. Table S3. Top miRNAs ranked by nominal (uncorrected) statistical significance of differential regulation between HA and LA DRGs following SNL nerve injury (subtraction method, HA_SNL1 pool excluded). miRNAs with significant diff-reg after FDR correction (Table 1) are set in italics. Table S4. Top miRNAs ranked by magnitude of differential regulation between HA and LA DRGs following SNL nerve injury (subtraction method). Table S5. miRNAs identified in rat DRGs that have not been previous reported in the rat (miRBase-v19). The ones with the prefix rnoH have homologs of the same number in other species (mostly the mouse). One was listed in the rat miRBase-v20. The ones numbered X1, X2 do not have homologs in any other species (sequence noted). [file 1744-8069-10-22-S1.docx]

13/11/13 for: Molecular Pain

**Sources of individual variability: miRNAs that predispose to neuropathic pain identified using genome-wide miRNA sequencing**

Kiran Kumar Bali *(1), Michael Hackenberg* (2), Avigail Lubin (3), Rohini Kuner (1) and Marshall Devor (3,**)

**Supplemental on-line material**

**Table S1:** Top 50 miRNAs ranked in order of abundance in the L4 and 5 DRGs of sham operated rats.

| **miRNA identifier** | **Rank** | **RPM (mean HA+LA sham)** |
| --- | --- | --- |
| rno-miR-10a-5p | 1 | 314,300 |
| rno-miR-143-3p | 2 | 66,575 |
| rno-miR-26a-5p | 3 | 59,791 |
| rno-miR-30a-5p | 4 | 51,250 |
| rno-miR-27b-3p | 5 | 30,346 |
| rno-miR-182 | 6 | 26,270 |
| *mmu-miR-486-5p* | *7* | *23,522* |
| rno-miR-127-3p | 8 | 23,924 |
| rno-miR-183-5p | 9 | 20,128 |
| rno-miR-146b-5p | 10 | 18,337 |
| rno-miR-181a-5p | 11 | 14,998 |
| rno-miR-22-3p | 12 | 13,515 |
| rno-let-7f-5p | 13 | 12,490 |
| rno-let-7c-5p | 14 | 11,091 |
| **rno-miR-30d-5p** | 15 | **10,205** |
| rno-miR-338-3p | 16 | 9,097 |
| rno-let-7i-5p | 17 | 9,168 |
| rno-miR-191a-5p | 18 | 8,134 |
| rno-miR-204-5p | 19 | 7,477 |
| rno-let-7a-5p | 20 | 6,137 |
| rno-miR-99b-5p | 21 | 5,334 |
| *rnoH-miR-148a-3p* | *22* | *4,326* |
| rno-miR-16-5p | 23 | 4,368 |
| rno-miR-146a-5p | 24 | 4,377 |
| rno-miR-100-5p | 25 | 4,033 |
| rno-miR-30e-5p | 26 | 3,689 |
| rno-miR-21-5p | 27 | 3,893 |
| rno-miR-26b-5p | 28 | 3,528 |
| rno-miR-125a-5p | 29 | 3,283 |
| **rno-miR-125b-5p** | 30 | **2,865** |
| rno-miR-99a-5p | 31 | 2,857 |
| rno-miR-24-3p | 32 | 2,543 |
| *rnoH-let-7g* | *33* | *2,313* |
| rno-miR-133a-3p | 34 | 2,026 |
| rno-miR-186-5p | 35 | 2,351 |
| rno-miR-23b-3p | 36 | 2,248 |
| rno-miR-92a-3p | 37 | 2,083 |
| rno-miR-151-3p | 38 | 2,003 |
| rno-miR-541-5p | 39 | 1,994 |
| rno-miR-101a-3p | 40 | 1,728 |
| *rnoH-miR-676-3p* | *41* | *1,754* |
| rno-miR-181c-5p | 42 | 1,690 |
| rno-miR-29a-3p | 43 | 1,649 |
| rno-miR-378a-3p | 44 | 1,486 |
| rno-miR-351-5p | 45 | 1,506 |
| rno-let-7b-5p | 46 | 1,485 |
| rno-miR-23a-3p | 47 | 1,395 |
| rno-miR-30c-5p | 48 | 1,302 |
| rno-miR-125b-2-3p | 49 | 1,212 |
| rno-miR-1-3p | 50 | 1,019 |

**Bold font**: *diff-reg* miRNAs (see Table 1).

*Italic font*: miRNAs novel in the rat, here designated rnoH.

**Table S2:** miRNAs nominally up- and down-regulated (p<0.05 before correction for multiple testing) after SNL nerve injury in HA (n=23) and LA rats (n=35; ranked by p-value). *SNL-reg* was calculated from RPM by the subtraction method: (SNL-sham)/(SNL+sham). None showed significant *SNL-reg* after FDR correction for multiple testing.

| **miRNA identifier** | ***SNL-reg*** | **Nominal**  **p-value*** | **rank** | **miRNA identifier** | ***SNL-reg*** | **Nominal**  **p-value*** |
| --- | --- | --- | --- | --- | --- | --- |
| **HA** |  |  |  | **LA** |  |  |
| rno-miR-449c-5p** | 1.18113342 | 0.00142656 | 1 | rno-miR-1224 | -0.704 | 0.00022 |
| rno-miR-1224 | -0.65751003 | 0.00161195 | 2 | rno-miR-99a-5p | -0.145 | 0.00189 |
| rno-miR-341*** | 0.42309693 | 0.01559552 | 3 | rno-miR-341 | 0.266 | 0.00339 |
| rno-miR-181c-3p | -0.15903512 | 0.01695948 | 4 | rno-miR-146b-5p | 0.844 | 0.00659 |
| rno-miR-106b-3p | 0.24509615 | 0.01748973 | 5 | rno-miR-155-5p | 0.435 | 0.00808 |
| rno-miR-28-5p | 0.20285537 | 0.02039028 | 6 | rno-miR-380-3p | 0.366 | 0.00874 |
| rno-miR-300-3p | 0.13403015 | 0.02106361 | 7 | rno-miR-758-3p | 0.214 | 0.00939 |
| rno-miR-708-3p | 0.20128190 | 0.02448413 | 8 | rno-miR-30a-5p | -0.072 | 0.01007 |
| rno-miR-99a-3p | -0.24362166 | 0.02707830 | 9 | rno-miR-493-3p | 0.314 | 0.01020 |
| rno-miR-30c-1-3p | -0.32367054 | 0.02711164 | 10 | rno-miR-203a-3p | 0.229 | 0.01067 |
| rno-miR-92b-3p | -0.36091105 | 0.02740073 | 11 | rno-miR-540-3p | 0.471 | 0.01111 |
| rno-miR-30d-5p | -0.12089224 | 0.02919076 | 12 | rno-miR-222-3p | 0.651 | 0.01254 |
| rno-miR-504 | -0.29611221 | 0.03606725 | 13 | rno-miR-379-3p | 0.402 | 0.01666 |
| rno-miR-27a-5p | 0.49292699 | 0.03633333 | 14 | rno-miR-449c-5p | 1.099 | 0.01741 |
| rno-miR-325-3p | 0.32890590 | 0.03799526 | 15 | rno-miR-351-5p | 0.115 | 0.02016 |
| rno-miR-124-3p | 0.11948155 | 0.03898249 | 16 | rno-miR-103-3p | -0.194 | 0.02056 |
| rno-miR-337-3p | 0.44355811 | 0.04058615 | 17 | rno-miR-221-5p | 0.719 | 0.02262 |
| rno-miR-20a-5p | 0.44294118 | 0.04078018 | 18 | rno-miR-582-3p | 0.474 | 0.02300 |
| rno-miR-195-3p | -0.40447944 | 0.04450323 | 19 | rno-miR-541-5p | 0.242 | 0.02390 |
| rno-miR-582-3p | 0.38464094 | 0.04476512 | 20 | rno-miR-30a-3p | -0.177 | 0.02423 |
| rno-miR-361-3p | -0.17213159 | 0.04501738 | 21 | rno-miR-409a-3p | 0.273 | 0.02872 |
| rno-miR-199a-5p | -0.20031742 | 0.04518613 | 22 | rno-let-7f-2-3p | 0.448 | 0.02927 |
| rno-miR-10a-3p | -0.45253262 | 0.04888007 | 23 | rno-miR-221-3p | 0.456 | 0.03035 |
|  |  |  | 24 | rno-miR-9a-3p | -0.146 | 0.03295 |
|  |  |  | 25 | rnoH-miR-676-3p | -0.219 | 0.03604 |
|  |  |  | 26 | rno-miR-6328 | 0.400 | 0.03677 |
|  |  |  | 27 | rno-miR-300-3p | 0.145 | 0.03744 |
|  |  |  | 28 | rno-miR-1839-5p | 0.169 | 0.03812 |
|  |  |  | 29 | rno-miR-223-3p | 0.488 | 0.03899 |
|  |  |  | 30 | rno-miR-21-3p | 0.870 | 0.04248 |
|  |  |  | 31 | rno-miR-378b | -0.331 | 0.04440 |
|  |  |  | 32 | rno-miR-92b-3p | -0.337 | 0.04503 |
|  |  |  | 33 | rno-miR-130b-3p | 0.422 | 0.04567 |
|  |  |  | 34 | rno-miR-21-5p | 0.786 | 0.04670 |
|  |  |  | 35 | rno-miR-337-3p | 0.411 | 0.04924 |

* based on 1-tailed t-tests.

** The only miRNA with significant SNL-reg after FDR correction

*** The only miRNA with significant regulation in the study by Li et al. [19].

**Table S3:** Top miRNAs ranked by nominal (uncorrected) statistical significance of differential regulation between HA and LA DRGs following SNL nerve injury (subtraction method, HA_SNL1 pool excluded). miRNAs with significant *diff-reg* after FDR correction (Table 1) are set in italics*.*

| **Rank** | **miRNA identifier**  **name** | **RPM (mean HA+LA sham)** | **Nominal**  **p-value** |
| --- | --- | --- | --- |
| ***1*** | ***rno-miR-125b-5p*** | ***3147.6*** | ***0.00001*** |
| *2* | *rno-miR-322-3p* | *30.1* | *0.00007* |
| *3* | *rno-let-7f-2-3p* | *9.9* | *0.00031* |
| ***4*** | ***rno-miR-30d-5p*** | ***10117.3*** | ***0.00057*** |
| *5* | *rno-miR-340-3p* | *10.2* | *0.00058* |
| *6* | *rno-miR-378b* | *49.4* | *0.00077* |
| *7* | *rno-miR-369-3p* | *12.8* | *0.00081* |
| *8* | *rno-miR-6331* | *12.8* | *0.00142* |
| ***9*** | ***rno-miR-379-5p*** | ***194.7*** | ***0.00151*** |
| 10 | rno-miR-132-3p | 16.1 | 0.00183 |
| *11* | *rno-miR-1839-5p* | *110.5* | *0.00266* |
| 12 | rno-miR-32-5p | 9.5 | 0.00325 |
| 13 | rno-miR-30a-5p | 61907.9 | 0.00376 |
| 14 | rno-miR-10b-5p | 74.4 | 0.00448 |
| 15 | rno-miR-504 | 42.1 | 0.00585 |
| *16* | *rno-miR-493-3p* | *73.1* | *0.00598* |
| 17 | rno-miR-10a-3p | 13.2 | 0.00650 |
| 18 | rno-miR-330-5p | 114.5 | 0.00789 |
| 19 | rno-miR-30a-3p | 327.3 | 0.00944 |
| 20 | rno-miR-142-3p | 36.1 | 0.00965 |
| 21 | rno-miR-141-3p | 10.8 | 0.01097 |
| 22 | rno-miR-107-3p | 146.5 | 0.01436 |
| 23 | rno-miR-221-5p | 20.4 | 0.01471 |
| 24 | rno-miR-30e-5p | 3587.5 | 0.01728 |
| 25 | rno-miR-16-5p | 4385.7 | 0.01881 |
| 26 | rno-miR-325-3p | 14.7 | 0.01942 |
| 27 | rno-miR-125a-5p | 3538.1 | 0.01948 |
| 28 | rno-miR-17-5p | 25.0 | 0.01979 |
| 29 | rnoH-miR-676-3p | 1840.7 | 0.02090 |
| 30 | rnoH-miR-148a-5p | 357.5 | 0.02143 |
| 31 | rno-miR-708-3p | 8.4 | 0.02217 |
| 32 | rno-miR-218a-5p | 178.4 | 0.02286 |
| 33 | rnoH-miR-149-5p | 147.3 | 0.02381 |
| 34 | rno-miR-144-5p | 43.0 | 0.02432 |
| 35 | rno-miR-1843-5p | 103.1 | 0.02518 |
| 36 | rno-miR-145-5p | 366.6 | 0.02547 |
| 37 | rno-miR-339-3p | 21.9 | 0.02672 |
| 38 | rno-miR-223-5p | 7.5 | 0.02746 |
| 39 | rno-miR-30c-1-3p | 14.1 | 0.02827 |
| 40 | rno-miR-31a-5p | 20.7 | 0.02858 |
| 41 | rno-miR-199a-5p | 304.9 | 0.03093 |
| 42 | rno-miR-598-3p | 91.0 | 0.03207 |
| 43 | rno-miR-101b-3p | 84.0 | 0.03407 |
| 44 | rno-miR-10b-3p | 18.3 | 0.03793 |
| 45 | rno-miR-184 | 12.6 | 0.03860 |
| 46 | rno-miR-382-5p | 36.6 | 0.04249 |
| 47 | rno-miR-376a-3p | 8.5 | 0.04647 |
| 48 | rno-miR-409a-3p | 50.9 | 0.04667 |
| 49 | rno-miR-203a-3p | 43.1 | 0.04796 |
| 50 | rno-miR-376b-3p | 20.7 | 0.04887 |
| 51 | rno-miR-758-3p | 95.4 | 0.05102 |
| 52 | rno-miR-103-3p | 917.6 | 0.05568 |
| 53 | rno-miR-27a-3p | 977.0 | 0.05618 |
| 54 | rno-miR-874-3p | 53.1 | 0.05942 |
| 55 | rno-miR-434-3p | 652.8 | 0.06015 |

**Table S4:** Top miRNAs ranked by magnitude of differential regulation between HA and LA DRGs following SNL nerve injury (subtraction method).

|  | **HA** **- LA**  *diff-reg* **>0** | | **HA - LA**  *diff-reg* **<0** | |
| --- | --- | --- | --- | --- |
| **Rank** | **miRNA identifier** | ***Diff-reg*** | **miRNA identifier** | ***Diff-reg*** |
| 1 | rno-miR-326-3p | 0.61 | rno-miR-221-5p | -0.61 |
| 2 | rno-let-7i-3p | 0.54 | rno-miR-184 | -0.55 |
| 3 | rno-miR-137-3p | 0.46 | rno-miR-130b-3p | -0.54 |
| 4 | rno-miR-378b | 0.46 | rno-miR-542-5p | -0.53 |
| 5 | rno-miR-133b-3p | 0.44 | rno-miR-31a-5p | -0.53 |
| 6 | rno-miR-340-3p | 0.43 | rno-miR-322-3p | -0.49 |
| 7 | rno-miR-30c-2-3p | 0.38 | rno-miR-10a-3p | -0.47 |
| 8 | rno-miR-345-5p | 0.36 | rno-miR-543-3p | -0.46 |
| 9 | rno-miR-32-5p | 0.36 | rno-miR-21-3p | -0.45 |
| 10 | rno-miR-138-5p | 0.35 | rno-miR-496-3p | -0.43 |
| 11 | rno-miR-345-3p | 0.33 | rno-miR-146b-5p | -0.40 |
| 12 | mmu-miR-486-5p | 0.31 | rno-miR-195-3p | -0.40 |
| 13 | rno-miR-134-5p | 0.30 | rno-let-7f-2-3p | -0.40 |
| 14 | rno-miR-24-1-5p | 0.29 | rno-miR-323-3p | -0.40 |
| 15 | rno-miR-29c-5p | 0.29 | rno-miR-129-2-3p | -0.40 |
| 16 | rno-miR-133a-3p | 0.28 | rno-miR-30b-5p | -0.38 |
| 17 | rno-miR-144-3p | 0.27 | rno-miR-148b-5p | -0.37 |
| 18 | rno-miR-9a-3p | 0.25 | rno-miR-30c-1-3p | -0.37 |
| 19 | rno-miR-144-5p | 0.25 | rno-miR-145-5p | -0.35 |
| 20 | rno-miR-1-3p | 0.25 | rno-miR-329-3p | -0.33 |
| 21 | rno-let-7d-3p | 0.24 | rno-miR-455-3p | -0.32 |
| 22 | rno-miR-129-5p | 0.22 | rno-miR-223-3p | -0.32 |
| 23 | rno-miR-325-3p | 0.22 | rno-miR-182 | -0.30 |
| 24 | rno-miR-20a-5p | 0.22 | rno-miR-380-3p | -0.30 |
| 25 | rno-miR-378a-3p | 0.22 | rno-miR-10b-3p | -0.29 |
| 26 | rno-miR-1249 | 0.21 | rno-miR-6331 | -0.29 |
| 27 | rnoH-miR-1247-5p | 0.21 | rno-miR-136-3p | -0.29 |
| 28 | rno-let-7c-5p | 0.20 | rno-miR-183-5p | -0.28 |
| 29 | rno-miR-652-3p | 0.19 | rno-let-7b-3p | -0.28 |
| 30 | rno-miR-101b-3p | 0.19 | rno-miR-379-3p | -0.28 |
| 31 | rno-let-7i-5p | 0.19 | rno-miR-1839-5p | -0.28 |
| 32 | rno-miR-145-3p | 0.18 | rno-miR-30c-5p | -0.28 |
| 33 | rno-miR-27a-5p | 0.18 | rno-miR-191a-5p | -0.28 |
| 34 | rnoH-miR-149-5p | 0.18 | rno-miR-199a-5p | -0.27 |
| 35 | rnoH-miR-676-3p | 0.18 | rno-miR-141-3p | -0.27 |
| 36 | rno-miR-196b-5p | 0.18 | rno-miR-541-5p | -0.27 |
| 37 | rno-miR-451-5p | 0.17 | rno-miR-298-5p | -0.27 |
| 38 | rno-miR-9a-5p | 0.17 | rno-miR-322-5p | -0.27 |
| 39 | rno-miR-341 | 0.16 | rno-miR-93-5p | -0.26 |
| 40 | rno-miR-210-3p | 0.15 | rno-miR-223-5p | -0.26 |
| 41 | rno-miR-382-5p | 0.15 | rno-miR-339-3p | -0.26 |
| 42 | rno-miR-181a-1-3p | 0.15 | rnoH-miR-148a-3p | -0.26 |
| 43 | rno-miR-329-5p | 0.14 | rno-miR-484 | -0.26 |
| 44 | rno-miR-190a-5p | 0.14 | rno-miR-497-5p | -0.25 |
| 45 | rno-miR-339-5p | 0.14 | rno-miR-21-5p | -0.25 |
| 46 | rno-miR-412-5p | 0.14 | rno-miR-487b-3p | -0.25 |
| 47 | rno-miR-592 | 0.13 | rno-miR-99b-5p | -0.24 |
| 48 | rno-miR-124-3p | 0.13 | rno-miR-25-3p | -0.24 |
| 49 | rno-miR-328a-3p | 0.12 | rno-miR-186-5p | -0.24 |
| 50 | rno-miR-99a-5p | 0.12 | rno-miR-26a-5p | -0.24 |
| 51 | rno-miR-3068-5p | 0.12 | rno-miR-28-3p | -0.24 |
| 52 | rno-miR-132-3p | 0.12 | rno-miR-199a-3p | -0.24 |
| 53 | rno-miR-598-3p | 0.12 | rno-miR-140-5p | -0.23 |
| 54 | rno-miR-6325 | 0.11 | rno-miR-3068-3p | -0.23 |
| 55 | rno-let-7f-1-3p | 0.11 | rno-miR-222-3p | -0.23 |

**Table S5:** miRNAs identified in rat DRGs that have not been previous reported in the rat (miRBase-v19). The ones with the prefix rnoH have homologs of the same number in other species (mostly the mouse). One was listed in the rat miRBase-v20. The ones numbered X1, X2 do not have homologs in any other species (sequence noted).

|  | **miRNA** | **RPM**  (mean of all sham pools) | **Note:** |
| --- | --- | --- | --- |
| 1 | rno-miR-X1-3p | 0.5 | TGGAGCTCTAACATGTCTGCC |
| 2 | rno-miR-X1-5p | 0.5 | AAAGACTGGGAGGGCTGCTCAGT |
| 3 | rno-miR-X2-3p | 1.4 | TGCTTTCGGAGTGTTCCTGTGC |
| 4 | rno-miR-X2-5p | 0.4 | CCAGGTCCACTCTGCTGAGCACT |
| 5 | rnoH-miR-744-3p | 183.3 |  |
| 6 | rnoH-miR-676-5p | 2.8 |  |
| 7 | rnoH-miR-676-3p | 1,840.7 |  |
| 8 | rnoH-miR-26a-2-3p | 9.3 |  |
| 9 | rnoH-miR-193b-3p | 20.9 |  |
| 10 | rno-miR-155-5p | 13.7 | in miRBase-v20 (not in -v19) |
| 11 | rnoH-miR-149-5p | 147.3 |  |
| 12 | rnoH-miR-148a-5p | 357.5 |  |
| 13 | rnoH-miR-148a-3p | 4,409.6 |  |
| 14 | rnoH-miR-1271 | 5.0 | not previously known in rodents |
| 15 | rnoH-miR-1247-5p | 13.1 |  |
| 16 | rnoH-let-7g | 2,582.7 |  |
| 17 | mmu-miR-486-5p | 23,522.4 |  |
| 18 | mmu-miR-486-3p | 7.6 |  |
